# Nanoencapsulated phase change material in a trapezoidal prism wall under the magnetic field effect for energy storage purposes

**DOI:** 10.1038/s41598-023-43394-2

**Published:** 2023-09-25

**Authors:** Obai Younis, Aissa Abderrahmane, Mohammad Hatami, Abed mourad, Kamel Guedri

**Affiliations:** 1 Department of Mechanical Engineering, College of Engineering in Wadi Alddawasir, Prince Sattam Bin Abdulaziz University, Wadi Alddawasir, Saudi Arabia; 2 Laboratoire de Physique Quantique de la Matière et Modélisation Mathématique (LPQ3M), University of Mascara, Mascara, Algeria; 3 Mechanical Engineering Department, Esfarayen University of Technology, Esfarayen, North Khorasan Iran; 4 Mechanical Engineering Department, College of Engineering and Islamic Architecture, Umm Al-Qura University, P.O. Box 5555, 21955 Makkah, Saudi Arabia

**Keywords:** Computational nanotechnology, Environmental, health and safety issues, Energy infrastructure, Mechanical engineering

## Abstract

Recently, Nano-encapsulated phase change materials (NEPCM) have attracted the attention of researchers due to their promising application in thermal management. This research investigates magnetohydrodynamic mixed convection of NEPCM contained within a lid-driven trapezoidal prism enclosure containing a hot-centered elliptical obstacle. The upper cavity wall is moving at a constant velocity; both inclined walls are cold, while the rest of the walls are insulated. The Galerkin Finite Element Method was used to solve the system's governing equations. The influence of Reynolds number (Re 1–500), Hartmann number (Ha = 0–100), NEPCM volumetric fraction φ (0–8%), and elliptical obstacle orientation α (0–3π/4) on thermal fields and flow patterns are introduced and analyzed. The results indicated that the maximum heat transfer rate is observed when the hot elliptic obstacle is oriented at 90°; an increment of 6% in the Nu number is obtained in this orientation compared to other orientations. Reducing Ha from 100 to 0 increased Nu by 14%. The Maximum value of the Bejan number was observed for the case of Ha = 0, α = 90° and φ = 0.08.

## Introduction

PCMs have been integrated with a variety of technical applications, most notably for thermal energy storage TES. PCMs have been used in a variety of applications, including building thermal management^1–3^, solar collectors^4^, thermal energy storage^5–8^ and other applications such as space equipment^9,10^, electronic cooling^11^, and the packaging industry^12^. Despite the fact that PCMs may store and release a large amount of energy when they change phases, they have a low thermal conductivity. As a result, numerous researchers^13–16^ have experimented with utilizing fins or metal foams to improve the heat transmission properties of PCM. Deng et al.^17^ conducted a thorough study of the melting performance of a finned heat sink integrated with a PCM. The findings show that the presence of fins significantly enhances the thermal performance of PCM-based heat sinks and that increasing the number of fins results in a lower working temperature, extending the temperature of electronic devices to an acceptable temperature range. Castell et al.^18^ an executed experimental study to evaluate the heat transfer rate by free convection in PCM modules with external vertical extending surfaces. Al-Mudhafar et al.^19^ suggested accelerating the PCM melting process in a shell and tube heat exchanger by employing Tee-shaped fins. The findings indicated that after 6 h, about 15% of the PCM in the case without fins melted, while after 3.5 h, the PCM melted completely in the case with tee fins. Dmitruk et al.^20^ investigated the use of spatially structured pin–fin metal alloy to enhance heat transmission inside paraffin PCM contained within a heat storage unit. They verified via repeated heating/cooling cycles that the pin–fin construction improved heat transmission inside the heat storage unit, thus lowering the temperature gradient within the heat accumulator. Tauseef‑ur‑ Rehman et al.^21^ investigated the performance of a high-porosity (97 percent) copper and nickel foam in a PCM-based heat sink (RT-54HC) under various heat loads. Copper foam with a 0.8 volume percentage of PCM was shown to decrease the base temperature by 26% when compared to nickel foam without PCM at 24 W. Lei et al.^22^ observed the influence of pore size distribution using Voronoi tessellations on thermal conductivity and phase change behavior of hierarchical foams/PCMs composites. Abbasov^23^ investigated the rate of heat transfer of open-cellular foam filled with PCM and having 12 solid tubular ribs in each cubic unit. It was demonstrated that metal foams with a greater radius to length ratio of the tubular rib might achieve better thermal conductivity. Thalmaier et al.^24^ examined the effect of adding metallic aluminum foam produced from recycled sawing chips on the thermal conductivity of paraffin. According to their findings, the thermal conductivity of the paraffin/metallic foam composite rose to 2.48 W/mK, and the thermal diffusivity grew to 9.1 m^2^/s, an increase of nearly tenfold in conductivity and sevenfold in thermal diffusivity, respectively. PCM leaking is an inherent drawback that limits the usage of PCM. The leakage of PCM takes place during various processes, such as undergoing a phase change and also due to the physical stresses of distribution. One way of preventing PCM leaking is encapsulating the PCM inside a shell to provide structural strength. Expanded graphite, Polyurethane, and silica fume^25–27^ are commonly sued materials for PCM encapsulating.

Nanoparticles have also been used in a number of experiments to improve the heat transmission and phase change of PCMs. This research introduces solid nanoparticles to PCM as additives to improve the medium's heat transfer properties^28–30^. Other researchers have experimented with dispersing NEPCM particles rather than metallic or non-metallic nanoparticles to improve classical heat transfer fluids' overall heat transfer capacities. In nano-encapsulation of PCM, the PCMs are encased in a nanometer-sized capsule shell and then scattered in a working fluid^31–34^. Tumirah Khadiran et al.^35^ developed and analyzed n-nonadecane nanocapsules for use in thermal energy storage. The primary discovery was that even after 1000 cycles of a thermal cycling test, the n-nonadecane nanocapsules maintain excellent thermal and chemical stability. The temperature and latent heat of freezing and melting of n-nonadecane nanocapsules, respectively, were found to be 30.2 °C, 82.0 J/g and 33.1 °C, 76.9 J/g. Valizadeh et al.^36^ produced and evaluated the Physico-chemical and thermal characteristics of AP25 nanocapsules as organic PCM for TES. They observed that encapsulation effectively worked as an additional protective screen to avoid leakage. Shuying Wu et al.^37^ investigated the phase change properties of NEPCM consisting of Lauric acid (LA) coated with carbon nanotubes (CNTs). According to their findings, the thermal conductivity and energy flow of new NEPCM are greater than those of pure LA at the same temperature. These findings strongly suggest the heat and mass transmission of LA by nano-encapsulation in CNTs.

Most of the studies on convective flow with NEPCM particles have focused on natural convection in various cavities. Free convection and entropy of NEPCM-water in a reverse T-shaped porous chamber fitted with corrugated baffles were investigated by Zidan et al.^38^ This research shows that increasing the Raleigh number causes the streamlines, velocity fields, and structural change in the phase change zone to intensify, but decreasing the Darcy number has the inverse result. The cooling performance of NEPCM loaded within an inclined square enclosure coupled with three Integrated Circuits was examined by Almutairi et al.^39^ According to the results, the inclined angle had no effect on the overall thermal performance at a Ra of 102. At Ra = 104, however, the inclination angle of 60 was the best value. Furthermore, when the volume fraction of NEPCM is 3%, and Ra is 104, overall heat transmission rises by 22–29%. Hajjar et al.^40^ discussed the free convection of NEPCM within a square cavity subjected to a time-periodic temperature. According to the findings, heat transmission in the enclosure is improved when a greater percentage of the NEPCM is utilized, with a fraction of 5% providing the best thermal performance. The average Nusselt number increased by 21%, and its highest value increased by 18.5% when the nanoparticle volume fraction increased from 2.5 to 5%. Ahmed et al.^41^ evaluated heat transmission and entropy of NEPCM owing to the interplay between the radiation and convection modes. The NEPCM/water mixtures flow within the prismatic enclosure with two designs (D1 and D2) dependent on the aspect ratio of the enclosure walls. The key findings revealed that changing the radiation parameter diminishes melting and solidification mechanisms. Furthermore, the use of NEPCMs improves heat transport significantly. Some studies focused on the forced convective flow of fluids with NEPCM particles. Ho et al.^42^ synthesized NEPCM-water suspension as the working fluid for heat removal from a microchannel heatsink (MCHS) and evaluated its heat transfer performance. According to their findings, including NECPM particles boost heat transmission and performance index by up to 70% and 45 percent, respectively. In another study^43^, they investigated the performance of NEPCM in a divergent MCHS. They stated that using NEPCMs with low heating loads at low Reynolds number flows might be beneficial to the thermal performance of the system. In comparison to pure water, the phase-transition heat transfer of NEPCM particles enhanced heat transmission by 82%. Using NEPCM slurry as a coolant, Rehman et al.^44^ designed a 3D numerical model to investigate the thermal performance and hydrodynamics aspects of the restricted slot jet impingement. They demonstrated that introducing NEPCM to the base fluid improves heat transmission by a significant amount. However, since slurry has a greater viscosity than the base fluid, it may cause a significant increase in system pressure loss, which rises with NEPCM particle concentration and jet Reynolds number. The cooling efficacy of a NEPCM-based heat transfer fluid in an MCHS with a hydraulic diameter of 2.6 mm was investigated by Joseph et al.^45^ Despite the fact that the NEPCM demonstrated a % improvement in heat transfer at 225 mL/min when compared to water, the pumping power rose by 17%. Nevertheless, the figure of Merit values demonstrates that the increase in pumping power is insignificant compared to the heat transfer improvement achieved for NEPCM. The thermal performance of a NEPCM mixture utilized as the working fluid in a pulsing heat pipe was examined by Heydarian et al.^46^. The research revealed that employing NEPCM paraffin dispersed in water as the working fluid raises heat transmission and lowers the pulsing heat pipe's thermal resistance. However, for NEPCM paraffin, there is an ideal concentration at which raising the concentration induces an increment in the thermal resistance of the pulsing heat pipe owing to the fluid's higher dynamic viscosity.

This research simulated and analyzed mixed convection flow and heat transfer of NEPCMs suspended in a Trapezoidal prism enclosure with a hot-centered elliptical hole. The current study focuses on the effects of Reynolds number, Hartmann number, elliptical hole inclination angle, and NEPCM nanoparticle concentration on flow and thermal field distributions, as well as Bejan and Nusselt numbers. To the authors’ knowledge, this investigation is unique.

## Physical model description

**Figure 1:**
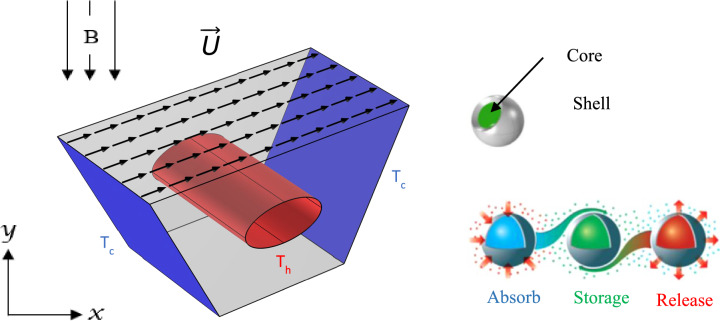
Physical problem (Designed by Photoshop CS v24.4.1.449).

The mixed convection of a nanoliquid and particles of a nanoencapsulated phase change material (NEPCM) is examined in a trapezoidal prism enclosure with a hot-centered elliptical obstacle and lid-driven top wall. The examined enclosure's structure is seen in Fig. 1 with boundary conditions.

The inclined side walls are cold and maintained at a constant temperature of Tc, whilst the elliptical surface has a temperature of Th with (Tc < Th). The top wall is sliding in the positive x-direction at a constant speed of U_0_. The enclosure is filled with NEPCM dispersed in water. A uniform magnetic field is applied. It is believed that the effects of Joule heating, displacement currents, radiation, and viscous dissipation are insignificant. Natural convection is approximated using the Boussinesq approximation in the buoyancy element of the momentum equation. Pressure adjustments have no effect on the density of nanoliquids. Temperature gradients, on the other hand, alter the density. The particles are distributed uniformly throughout the host fluid, and dynamic and thermal equilibrium between the nano-additives and the base fluid is established. Table 1 summarizes the thermophysical parameters of the components used to manufacture the nano-additives and the base fluid.

**Table 1:** Thermophysical properties of the shell and core of the NEPCMs and liquid^44^.

Material: application	$\beta \left({\mathrm{K}}^{-1}\right)$	$Cp(\mathrm{kJ}/\mathrm{kg} \, \mathrm{K})$	$k (\mathrm{W}/\mathrm{mK})$	$\rho \left(\mathrm{kg}/{\mathrm{m}}^{3}\right)$	$\upmu *{10}^{-6 }\left(\frac{\mathrm{kg}}{\mathrm{ms}}\right)$
Polyurethane: shell	$17.28\times {10}^{-5}$	$1.3177$	$0.025$	786	797
Nonadecane: core	$50\times {10}^{-5}$	$2.037$	$0.19$	721	
Water: base fluid	$21\times {10}^{-5}$	$4.179$	$0.613$	$997.1$	

## Governing equations

Free and forced convection flow happens as a result of buoyancy forces and top wall movement, respectively. Combining the two results in mixed convection. The flow of liquid and NEPCM is 3D, steady-state, and incompressible. For linear density changes, the Boussinesq approximation is also considered^45^:

1 $$\frac{\partial u}{\partial x}+\frac{\partial v}{\partial y}+\frac{\partial w}{\partial \mathrm{z}}=0$$

2 $${\rho }_{m}\left(u\frac{\partial u}{\partial x}+v\frac{\partial u}{\partial y}+w\frac{\partial u}{\partial z}\right)=-\frac{\partial P}{\partial x}+{\mu }_{m}\left(\frac{{\partial }^{2}u}{\partial {x}^{2}}+\frac{{\partial }^{2}u}{\partial {y}^{2}}+\frac{{\partial }^{2}u}{\partial {z}^{2}}\right)$$

3 $${\rho }_{m}\left(u\frac{\partial v}{\partial x}+v\frac{\partial v}{\partial y}+w\frac{\partial v}{\partial z}\right)=-\frac{\partial P}{\partial y}+{\mu }_{m}\left(\frac{{\partial }^{2}v}{\partial {x}^{2}}+\frac{{\partial }^{2}v}{\partial {y}^{2}}+\frac{{\partial }^{2}v}{\partial {z}^{2}}\right)+(\rho \beta {)}_{m}\left(T-{T}_{C}\right)g.$$

4 $${\rho }_{m}\left(u\frac{\partial w}{\partial x}+v\frac{\partial w}{\partial y}+w\frac{\partial w}{\partial z}\right)=-\frac{\partial P}{\partial x}+{\mu }_{m}\left(\frac{{\partial }^{2}w}{\partial {x}^{2}}+\frac{{\partial }^{2}w}{\partial {y}^{2}}+\frac{{\partial }^{2}w}{\partial {z}^{2}}\right)-\frac{{\sigma }_{nf}{B}_{0}^{2}v}{{\rho }_{nf}}$$

5 $$\left(u\frac{\partial }{\partial x}\left({\left(\rho {C}_{P}\right)}_{m}T\right)+v\frac{\partial }{\partial y}\left({\left(\rho {C}_{P}\right)}_{m}T\right)+w\frac{\partial }{\partial z}\left({\left(\rho {C}_{P}\right)}_{m}T\right)\right)={k}_{m}\left(\frac{{\partial }^{2}T}{\partial {x}^{2}}+\frac{{\partial }^{2}T}{\partial {y}^{2}}+\frac{{\partial }^{2}T}{\partial {z}^{2}}\right).$$

The dimensionless parameters are given as^45^.

6 $$\begin{array}{cc}& (X,Y)=\left(\frac{x}{r},\frac{y}{r}\right),(U,V)=\left(\frac{u}{r\omega },\frac{v}{r\omega }\right),\theta =\frac{T-{T}_{C}}{{T}_{H}-{T}_{C}},p=\frac{P}{{\rho }_{bf}{r}^{2}{\omega }^{2}}\\ & \end{array}$$

we may render the governing equations dimensionless^45^.

7 $$\frac{\partial U}{\partial X}+\frac{\partial V}{\partial Y}+\frac{\partial W}{\partial Z}=0$$

8 $$\left(\frac{{\rho }_{m}}{{\rho }_{bf}}\right)\left(U\frac{\partial U}{\partial X}+V\frac{\partial U}{\partial Y}+W\frac{\partial U}{\partial Z}\right)=-\frac{\partial p}{\partial X}+\frac{1}{R{e}_{bf}}\left(\frac{{\mu }_{m}}{{\mu }_{bf}}\right)\left(\frac{{\partial }^{2}U}{\partial {X}^{2}}+\frac{{\partial }^{2}U}{\partial {Y}^{2}}+\frac{{\partial }^{2}U}{\partial {Z}^{2}}\right)$$

9 $$\begin{array}{cc}& \left(\frac{{\rho }_{m}}{{\rho }_{bf}}\right)\left(U\frac{\partial V}{\partial X}+V\frac{\partial V}{\partial Y}+W\frac{\partial V}{\partial Z}\right)=-\frac{\partial p}{\partial Y}+\frac{1}{R{e}_{bf}}\left(\frac{{\mu }_{m}}{{\mu }_{bf}}\right)\left(\frac{{\partial }^{2}V}{\partial {X}^{2}}+\frac{{\partial }^{2}V}{\partial {Y}^{2}}+\frac{{\partial }^{2}V}{\partial {Z}^{2}}\right)+\frac{G{r}_{bf}}{{\rho }_{bf}{}^{2}}\left(\frac{(\rho \beta {)}_{m}}{(\rho \beta {)}_{bf}}\right)\theta \\ & \end{array}$$

10 $$\left( {\frac{{\rho_{m} }}{{\rho_{bf} }}} \right)\left( {U\frac{\partial W}{{\partial X}} + V\frac{\partial W}{{\partial Y}} + W\frac{\partial W}{{\partial Z}}} \right) = - \frac{\partial p}{{\partial Y}} + \frac{1}{{Re_{bf} }}\left( {\frac{{\mu_{m} }}{{\mu_{bf} }}} \right)\left( {\frac{{\partial^{2} W}}{{\partial X^{2} }} + \frac{{\partial^{2} W}}{{\partial Y^{2} }} + \frac{{\partial^{2} W}}{{\partial Z^{2} }}} \right) - \frac{{\sigma_{hnf} }}{{\sigma_{f} }}Ha^{2} {\text{W}}$$

11 $$\left(U\frac{\partial }{\partial X}(Cr\theta )+V\frac{\partial }{\partial Y}(Cr\theta )\right)=\frac{1}{{\mathrm{Re}}_{bf}P{r}_{bf}}\left(\frac{{k}_{m}}{{k}_{bf}}\right)\left(\frac{{\partial }^{2}\theta }{\partial {X}^{2}}+\frac{{\partial }^{2}\theta }{\partial {Y}^{2}}\right)$$

The following is how the dimensionless numbers are expressed^42^:

12 $$v_{bf} = \frac{{\mu_{bf} }}{{\rho_{bf} }},\alpha_{bf} = \frac{{k_{bf} }}{{\left( {\rho c_{p} } \right)_{bf} }},Pr = \frac{{v_{bf} }}{{\alpha_{bf} }},Re_{bf} = \frac{{\omega r^{2} }}{{v_{bf} }},andGr_{bf} = \frac{{g\beta_{bf} {\Delta }Tr^{3} }}{{v_{bf} ^{2} }},Ha = LB\sqrt {\frac{{\sigma_{hnf} }}{{\mu_{hnf} }}} {.}$$

Additionally, the mixture's heat capacity is expressed to that of the water (Cr).

13 $$Cr=\frac{{\left(\rho {C}_{P}\right)}_{m}}{{\left(\rho {C}_{P}\right)}_{bf}}=1-\varphi +\lambda \varphi +\frac{\varphi }{\chi }f$$

With the NEPCM core's latent heat $\lambda$, which is determined using the following equation.

14 $$\lambda =\frac{\left({C}_{{p}_{c,1}}+l{C}_{{p}_{s}}\right){\rho }_{c}{\rho }_{s}}{\left({\rho }_{s}+l{\rho }_{c}\right){\left(\rho {C}_{P}\right)}_{bf}}$$

Additionally, at Eq. (14) $\chi$ is the ratio of a rise in the temperature of the liquid to the energy stored as latent heat in the core:

15 $$\chi =\frac{{C}_{P,bf}}{{h}_{sf}/{T}_{Mr}}\frac{{\rho }_{bf}\left({\rho }_{s}+l{\rho }_{c}\right)}{\left({\rho }_{s}{\rho }_{c}\right)}$$

Additionally, the variable f in Eq. (14) denotes the non-dimensional fusion function derived from the following equation.

16 $$f=\frac{\pi }{2}\mathrm{sin}\left(\frac{\pi }{\delta }\left(\theta -{\theta }_{f}+\frac{\delta }{2}\right)\right)\times \left\{\begin{array}{l}0\text{ if }\theta <{\theta }_{f}-\frac{\delta }{2}\\ 1\text{ if }{\theta }_{f}-\frac{\delta }{2}<\theta <{\theta }_{f}+\frac{\delta }{2}\\ 0\text{ if }\theta <{\theta }_{f}+\frac{\delta }{2}\end{array}\right.$$

17 $$\delta =\frac{{T}_{Mr}}{\Delta T},\text{ and }{\theta }_{f}=\frac{{T}_{f}-{T}_{C}}{\Delta T}$$

If the nanoliquid temperature is greater than the NEPCM core melting temperature $\left(T>{T}_{f}+\frac{{T}_{Mt}}{2}\right)$ or less than the core solidification temperature $\left(T<{T}_{f}-\frac{{T}_{\Delta u}}{2}\right.$), the last term of Eq. (9) is zero, and the values of Cr decrease.

The heat transfer rate can be calculated using the Nusselt number, which is as follows:

18 $$N{u}_{loc}=\frac{{k}_{m}}{{k}_{bf}}\frac{\partial \theta }{\partial n}, N{u}_{\text{Ave }}=\int N{u}_{loc}(n)dn.$$

The total entropy generation $S_{tot}$ is determined in dimensionless form as follows^26^:

19 $$S_{tot} = S_{ht} + S_{ff} + S_{mf}$$

where *S*_*ht*_*, S*_*ff*_*, S*_*mf*_ are the entropy generation due to the heat transfer, fluid shear, and magnetic force, respectively.

The relation for the Bejan number is given below.

20 $$Be=\frac{{S}_{ht}}{{S}_{tot}}$$

A polyurethane shell surrounds a nonadecane core in nano-encapsulated PCM. Thermophysical characteristics of NEPCM may be presented by considering both the core and thermophysical shell properties.:

21 $${\rho }_{n}=\frac{(1+l){\rho }_{c}{\rho }_{s}}{{\rho }_{s}+l{\rho }_{c}}$$

The specific heat capacity of the NEPCM core $\left({C}_{p,c}\right)$ in the phase change temperature range is :

22 $$\begin{array}{cc}{C}_{p,c}=& {C}_{p}c,t+\left\{\frac{\pi }{2}\left(\frac{{h}_{sf}}{{T}_{Mr}}-{C}_{pc,l}\right)\left(\mathrm{sin}\pi \frac{T-\left({T}_{f}-{T}_{Mr}2\right)}{{T}_{Mr}}\right)\right\}\\ & \times \left\{\begin{array}{c}0\text{ if }T<{T}_{f}-\frac{{T}_{Mr}}{2}\\ 1\text{ if }{T}_{f}-\frac{{T}_{Mr}}{2}<T<{T}_{f}+\frac{{T}_{Mr}}{2}\\ 0\text{ if }T<{T}_{f}+\frac{{T}_{Mr}}{2}\end{array}\right.\end{array}$$

The thermal expansion coefficient and specific heat capacity of NEPCM can be introduced as follows:

23 $${C}_{p,n}=\frac{\left({C}_{p,c}+l{C}_{s}\right){\rho }_{c}{\rho }_{s}}{\left({\rho }_{s}+l{\rho }_{c}\right){\rho }_{n}}$$

24 $$\begin{array}{cc}& \\ & {\beta }_{n}={\beta }_{c}+\left(\frac{{\beta }_{s}-{\beta }_{c}}{2}\right)\left(1-\frac{l{\rho }_{s}}{{\rho }_{c}}\right)\end{array}$$

By taking into account the thermophysical characteristics of water, NEPCM nanoparticles, and NEPCM volume fraction, the thermophysical characteristics of the water-NEPCM combination may be classified in Table 1. The following is a list of the thermophysical equations for the mixture, which include its density, specific heat capacity, and thermal expansion coefficient:

25 $$\begin{array}{cc}& {\rho }_{m}=\left(1-\varphi \right){\rho }_{bf}+\varphi {\rho }_{n}\\ & \end{array}$$

26 $${C}_{{P}_{m}m}=\frac{(1-\varphi ){\rho }_{f}{C}_{Pbf}+\varphi {\rho }_{n}{C}_{{P}_{,n}}}{{\rho }_{m}}$$

27 $${\beta }_{m}=\frac{(1-\varphi ){\rho }_{bf}{\beta }_{bf}+\psi {\rho }_{n}{\beta }_{n}}{{\rho }_{m}}$$

## Validation and mesh evaluation

**Figure 2:**
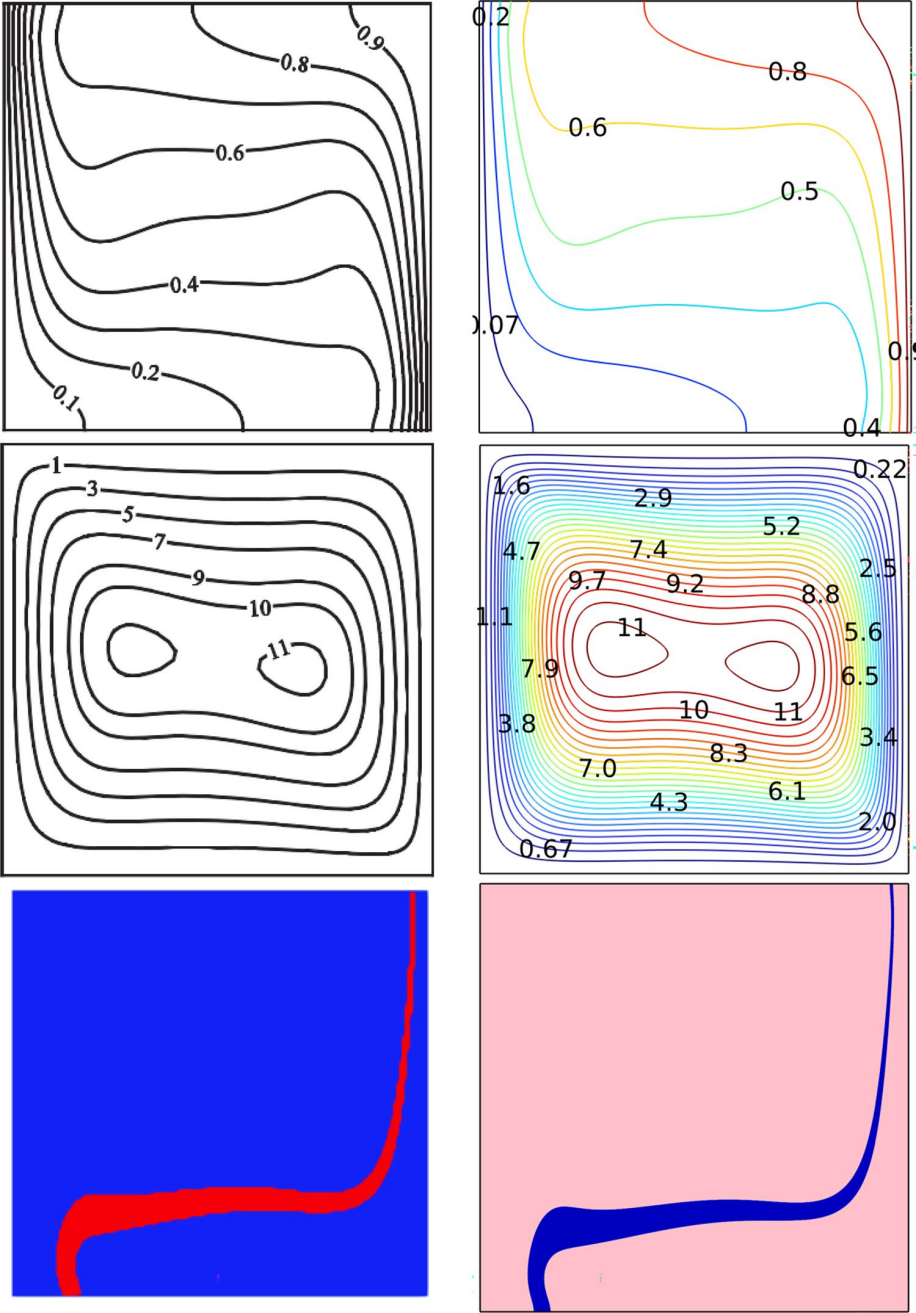
Comparison of current work with that of literature^46^.

The system of governing equations exposed to the above-mentioned boundary conditions is solved by employing the Galerkin-finite element technique. This technique employs the weighted residual method to transform the non-linear partial differential equations into a linear system of equations. Figure 2 represents the meshing of the computational domain employed in the current assessment. The results of the validation are shown in Table 2. For the validation, the (Nu) on the hot surface at (Re = 100, Ha = 0, ϕ = 4%) were utilized. The findings illustrate that the grid size of 23,362. Validation of the current findings was accomplished by numerical studies. In this context, Results produced from the current work's model are compared with those published in Ghalambaz et al.^46^, as shown in Fig. 2.

**Table 2:** Grid independence test for Re = 100, Ha = 0, ϕ = 4%.

No. of elements	1632	2302	$3486$	8987	**23,362**
${\Psi }_{\mathrm{max}}$	−33.782	−33.736	$-33.714$	−33.673	**−33.673**
$N{u}_{a}$	7.8673	7.8078	$7.8990$	7.8817	**7.8815**

## Results and discussion

**Figure 3:**
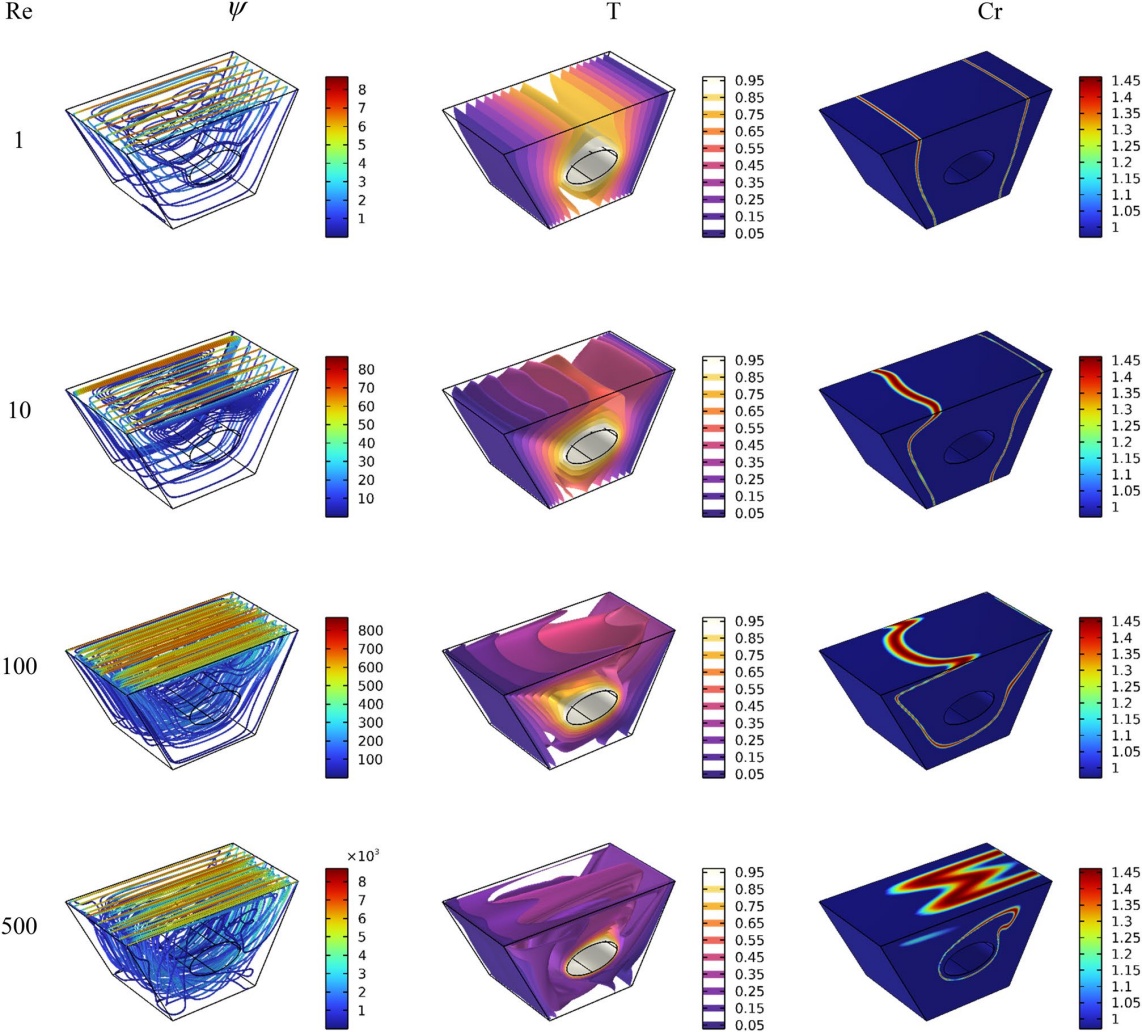
Re number influence on streamlines and isotherms surfaces and heat capacity ratio for Ha = 0, and φ = 5% (Designed by COMSOL Multiphysics 5.6).

**Figure 4:**
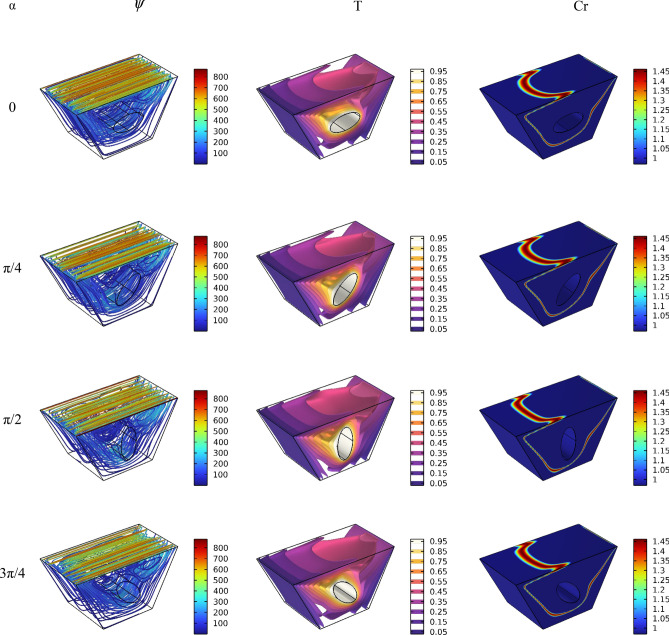
the effect of the elliptical obstacle orientation on streamlines and isotherms surfaces and heat capacity ratio for Re = 100, Ha = 0, and φ = 5% (Designed by COMSOL Multiphysics 5.6).

**Figure 5:**
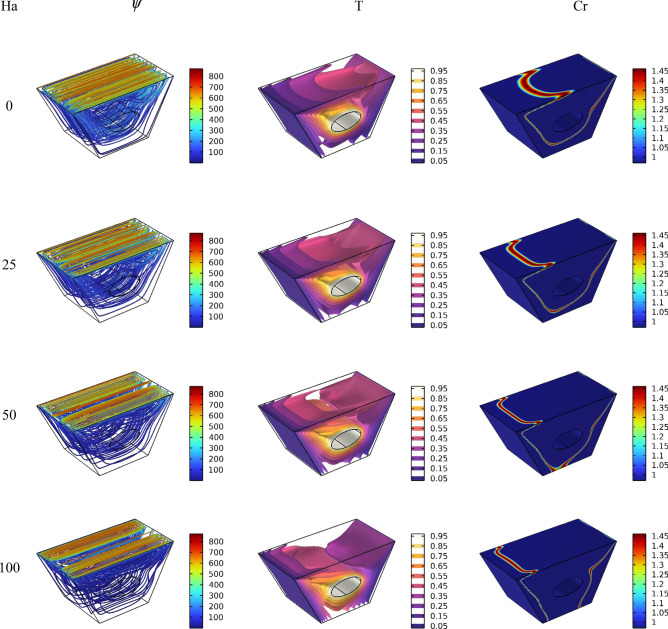
Ha number influence on streamlines and isotherms surfaces and heat capacity ratio for Re = 100, and φ = 5% (Designed by COMSOL Multiphysics 5.6).

**Figure 6:**
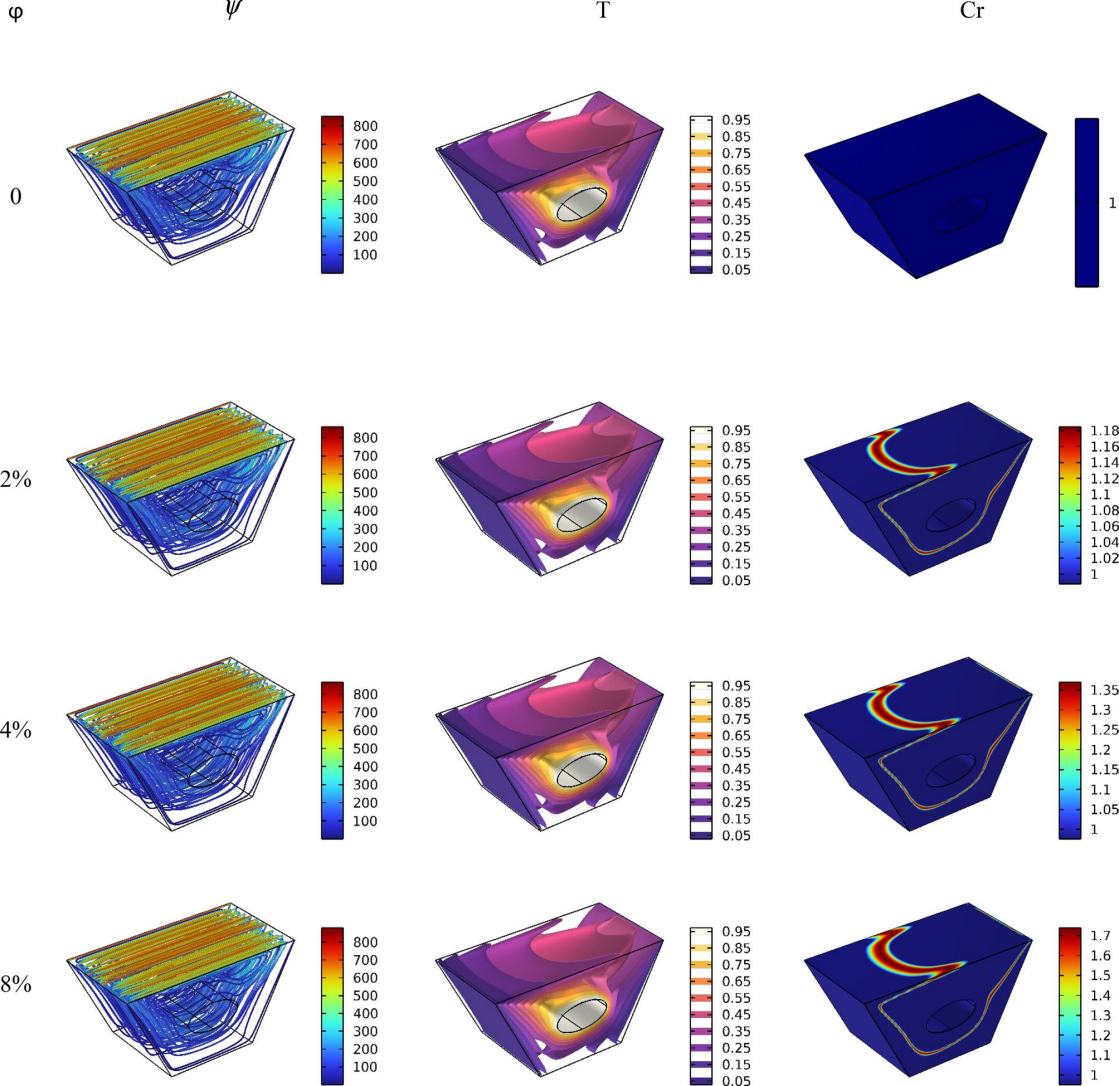
ϕ number influence on streamlines and isotherms surfaces and heat capacity ratio for Re = 100, and Ha = 0 (Designed by COMSOL Multiphysics 5.6).

**Figure 7:**
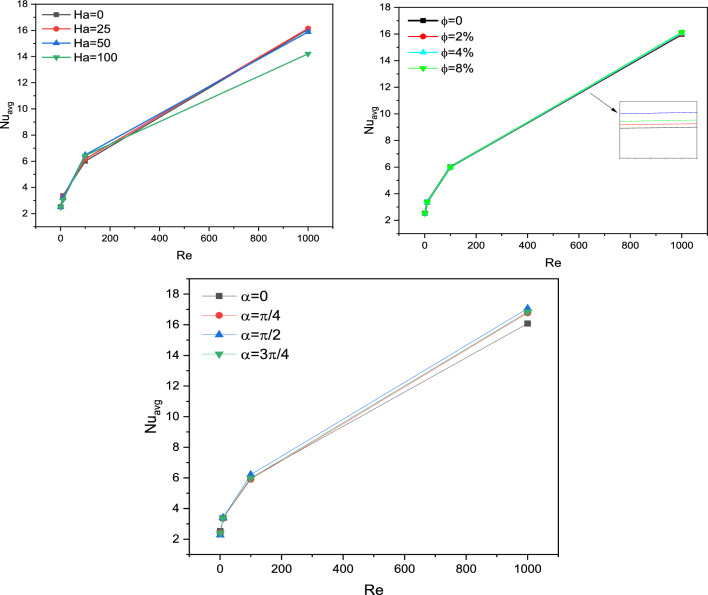
Nu _avg_ for different parameters.

**Figure 8:**
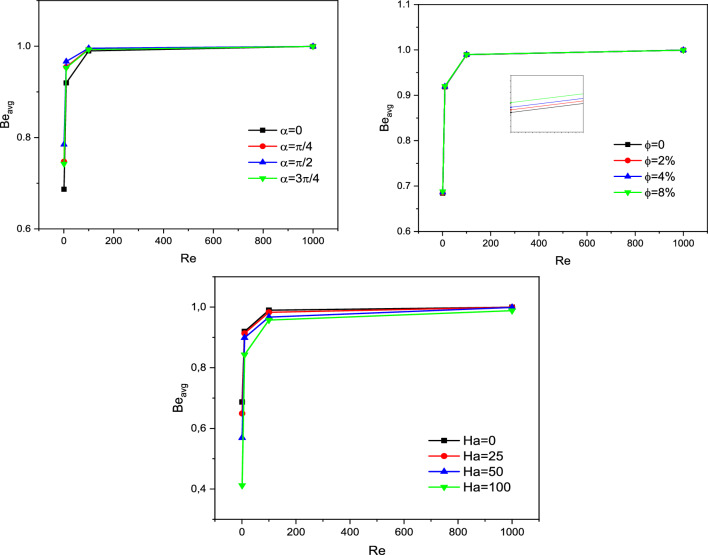
Bejan number for different parameters.

After the mesh independence study and validation of the current numerical code, the effect of parameters on the results is discussed. Since the problem is related to mixed convection in a lid-driven trapezoidal prism filled by NEPCM under the magnetic effect and a hot elliptic hole, the parameters considered as Hartmann number (Ha), nanoparticles concentration (ϕ), and elliptic inclined angle (α) and Reynolds number (Re). The results are streamlines, Entropy generation, temperature contours and liquid fraction, as well as the Nusselt and Bejan numbers. Figure 3 shows the main contours of streamline, temperature and entropy at different Reynolds numbers. As seen, increasing the Re caused more turbulence in streamlines and made more non-uniform temperature contours around the elliptic; actually, at high Re numbers, due to more heat transfer, the region of maximum temperature around the elliptic is reduced, and more heat is transferred to far regions, so the entropy is also increased at these high Reynolds numbers at the top of the enclosure. The elliptic inclined angle (α) is another main parameter that affects the outcomes, and its changes at four degrees (0, π/4, π/2, 3π/4) are depicted in Fig. 4. As seen, by rotating the elliptic, the heat transfer process improved due to flow patterns around the elliptic inconfirmence by the lid derived above the surface. When the rotation angle is 90°, due to the vertical shape of the elliptic, it has more heat transfer in the geometry. Figure 5 demonstrates the graphical results of the Hartmann number effect when the rotation angle is 0°. Due to the negative effect of the magnetic source on the streamlines and natural convection, it is observed that entropy generation is decreased, and consequently, the heat transfer is decreased. To find the effect of NEPCM volume fractions, Fig. 6 is depicted for the 3D contours. Actually, a greater nanoparticle volume fraction makes more heat transfer absorbed by NEPCM and consequently increases entropy generation. The effect of these parameters on the Results of average Nusselt number (Nu_ave_) and average Bejan number (Be_ave_) is presented in Figs. 7 and 8, respectively. As described above, increasing the Re makes an improvement in Nu and Be numbers in all cases. Figure 7 shows that reducing the Ha and increasing the ϕ are keynotes of Nu improvements, and in most Re numbers for the α = 90°, maximum Nu numbers were observed. Since the Bejan number is the relation of entropy generated by heat transfer to total generated entropy, Fig. 8 confirms that for Ha = 0, α = 90° and ϕ = 0.08, the maximum values of average Bejan numbers will occur.

## Conclusion

The current research discusses the magnetohydrodynamic mixed convection of NEPCM inside a trapezoidal lid-driven cavity containing a hot elliptic obstacle. The forced convection results from the movement of the upper cavity wall with constant velocity, while the natural convection is due to the temperature difference between the cold inclined walls and the hot obstacle. The Galerkin Finite Element Method (GFEM) was employed to address the equations governing the studied MHD mixed convection system. The influence of Re, Ha, φ, and α are reported and analyzed. Upon the above reported and discussed results, the following points could be concluded:

- Increasing the Re caused the region of maximum temperature around the hot elliptic to be reduced, and hence more heat is transferred to far regions. Consequently, the entropy is also increased at these high Reynolds numbers at the top of the enclosure.
- Changing the hot elliptic orientation enhanced the heat transfer process due to the reason that the flow patterns around the elliptic are inconfirmenced by the lid derived above the surface.
- The maximum heat transfer rate is observed when the rotation angle was 90°; an increment of 6% in the Nu number is obtained in this orientation compared to other orientations.
- Increasing NEPCM volume fraction enhanced the heat transfer rate absorbed by NEPCM, and consequently, the entropy generation was increased.
- Reducing Ha from 100 to 0 increased Nu by 14%.
- The Maximum value of the Bejan number was observed for the case of Ha = 0, α = 90° and ϕ = 0.08.
- Regarding heat transfer rate, it could be concluded that the rate of heat transfer reached its maximum under the following conditions: Re = 100, Ha = 0, α = 90°, and ϕ = 0.08.

## Data Availability

The datasets used and/or analysed during the current study available from the corresponding author on reasonable request.
